# Extra Supplement—The Nursing Mirror

**Published:** 1889-03-30

**Authors:** 


The Nursing Mirror.

All Contributions for this Supplement should be addressed "The Nursing Mirror," care of The Editor, 38, Old Jewry, E.C.

### En Passant.

A NURSES' SAVINGS FUND.—"An Army Sister's" suggestions have met with some support. Sixty names in approval of her scheme have been received, whilst about ten express their disapproval. The matter is now in the hands of the Pension Fund, who will, we feel confident, be willing, if possible, to meet the views thus expressed.

ASYLUM ATTENDANTS.—We are accused of having neglected our numerous readers in the asylums, and of not having devoted sufficient space to their interests. Perhaps the reason is that we have but few amongst them who are our regular correspondents, though the article on "The Care of the Insane," which appeared a fortnight ago, has called forth many letters. A nurse, late of Colney Hatch, has sent us a pamphlet called "Work in the Wards," by the Rev. H. Hawkins, which, she says, she found of the greatest interest and use. The whole motto of the book is "Not with eye-service," and the lesson it tries to inculcate is that impartiality, example, and influence can do much for the patients, though they are not ordered by the rules. We hope to commence next week a most interesting paper by Dr. Duncan Greenlees, on the "Nursing of the Insane."

WHAT NURSES SHOULD KNOW.—The young are proverbially intolerant, and surely the writers in the *Medical Press* must be very young to be so afraid of nurses. They are always fretting themselves about the proper status of nurses and the proper amount of intelligence that these women should possess. In a late article on "The Instruction of Nurses," it is said to be self-evident that the nursing will be no better in a case of a broken leg, because the nurse knows it is a comminuted fracture of the tibia. This is nonsense; if a nurse does not know what part is injured, how is she to see that proper rest of that part is maintained? The most awful accusation brought against the Catholic Church is that it fostered ignorance in the middle ages; but surely science ought to know better than to believe that good can ever arise from limiting the human understanding. "I do like a nurse to obey me pleasantly," said a bullying young doctor. "Then ask her pleasantly," was the prompt reply of his chief. Because women have undertaken the many difficult and trying duties of nursing is no reason for confining their thoughts solely to the sordid side of their labours; and to use phrases such as are quoted in the article in question is to show a lack of courtesy, and of that chivalric feeling which we thought nurses, even above other women, should inspire in all true men. A nurse has to confine her duties to details, but if she takes an interest in the treatment of her cases, so long as she does not interfere with the treatment, it is better for her patients. The loyalty and fealty a nurse feels towards a doctor for whom she works, and who trusts her and treats her fairly, is the best spirit a surgeon can rouse in his handmaid in the art of healing. Such a spirit is not likely to be roused by those who are jealous of a nurse, who are constantly suspicious of her, or who correct her in the vulgar manner admired by the writer of the article in question. The most implicit obedience we have ever seen was given by a well-educated intellectual woman who nursed for a celebrated surgeon for many years; and the surgeon in return always trusted this nurse and treated her with consideration. "I cannot do it unless I have my little sister," he would say, when asked to operate in a private house.

A CLUB-ROOM FOR NURSES.—We hear that The Hospitals Association, in conjunction with the Pension Fund, contemplate providing a room in a central position, where nurses may meet their friends. A reference library of books, dealing with hospital, nursing, and general topics, will also be provided.

THE SOUTHAMPTON INSTITUTE.—The twenty-first annual report of the Hampshire Nurses' Institute tells a satisfactory tale, in spite of the enlargement of the home last year, which cost £298. The nurses earned £753 last year, and received as wages £313. The staff of nurses numbers 17, and the probationers are trained at Addenbrooke's Hospital. The district nurses—day and night—have paid 6,658 visits, and the committee express their appreciation of the devotion of these nurses, and of the excellent services rendered by Mrs. Varian, the matron.

WORCESTER CITY INSTITUTION.—We have received the annual report of the Worcester City and County Institution for Trained Nurses, and perused it with pleasure. The objects of the institution are to provide a home for thoroughly-trained nurses, to be employed in nursing both among the poor and in private families, and to supply nurses to dispensaries, boards of guardians, local district nursing associations, and other charitable institutions. The deficiency of £54 with which the institution began last year has been converted into a balance of £76, which is greatly to the credit of the matron, Miss Holloway. There are thirty nurses and four probationers on the staff; they earned over £1,000 last year, and received as wages £800.

COMBINATION OF PRIVATE NURSES.—It has been suggested to us that we should act as treasurer for those nurses who desire to combine to secure their own advantage; but before money is sent to us it would be wise to test the general wish by inviting all private nurses who approve the scheme to send their name and address on a postcard, headed "Combination," to The Editor, The Lodge, Porchester Square, London, W. If 200 names are thus received, we shall be glad to arrange for these nurses to meet together, free of expense to themselves, in order that an accurate idea may be formed of what can be done for the interests of private nurses as a body. It would be well if nurses would state on the postcards what week day and what hour would be most convenient to them for such a meeting.

HEROISM.—A correspondent doubts whether we headed the paragraph about the Melbourne nurses in our issue of March 9th correctly. She considers that the nurses did their duty, but that heroism consists in something higher than this—in some voluntary danger accepted which is over and beyond our duty. We are very glad when our correspondents take up subjects, which are not purely nursing, and turn their thoughts for awhile to other things. Looking first to "Webster's Dictionary," we find heroism described as contempt of danger from noble devotion to some great cause. Carlyle defines heroism as the dazzling and glorious concentration of courage. Hare says it is the self-devotion of genius manifesting itself in action. But our correspondent must have been thinking of Charles Kingsley's beautiful essay on "Heroism," where he says it is the essence of self-sacrifice, and therefore of heroism, that it should be voluntary; a work of supererogation, at least, towards society and man; an act to which the hero or heroine is not bound by duty, but which is above, though not against, duty.

### Nurses on their Travels.

Another Letter from Madeira.

After weeks of fine weather, our Hospital Sunday (February 26th) was very wet. We had such an interesting account of the Funchal Seamen's Hospital, I determined to go and see it. I went, but the nurse-in-charge told me she had a bad fever case, and orders to admit no visitors. Better luck next time. The collections altogether came to £21; not bad, but more wanted. Sunday here is the grand parade of the lame, the halt and the blind. They line the roads, and assail passers-by with piteous whines and gestures. Very wretched the poor creatures look; and, for the size of the town, there seem a great many blind. These are the only adult beggars I have noticed, though the children all swarm after one, screeching "*reis-een, reis-een!*" and even babies in arms are taught to hold out their little hands.

My life here is one campaign against flies. They do not trouble the healthy much, but are a perfect pest to invalids. The first thing I say on making a fresh acquaintance is, "Can you tell me anything good" (of course I mean bad) "for flies?" I was told eucalyptus leaves will keep them away. I hung them up till the sick-room looked like Christmas, only to find the flies resting in dozens on them all night. Then we tried the oil in saucers, but they buzzed round in derision. "They can stand any smell we can, and a great many we cannot," says my patient. Next we got a trap, with a double covering of wire gauze, baited with sugar and vinegar: when full it is put in the oven a minute, and then the corpses are shaken out, quite crisp. Hundreds perish in it, but it is not much real good; two come to each funeral. It affords my patient satisfaction, however, when a few score of his tormentors get baked. My latest device is to unhook the fly paper, where they mostly congregate at night, shake it well in the open air, and then go round with a whisk and knock down others. Unless one is very quick, the cold night air and the shaking wakes them, and they fly back in a cloud. Can any reader of The Hospital help me? I heard great complaints of this sort during the Egyptian war.

The native servants here have elected me house doctor, and bring me their cut fingers, headaches, and stomach-aches to treat. My first patient was one of the hammock-bearers, who was starting off, carrying a letter, with a great cut across his finger dripping with blood. Pleasant for the lady the letter was going to! I dressed it, and have since had other jobs. I wish I had brought some common strapping. I miss my Pond's extract, too; I rashly presented it to a young lad who had helped make time pass pleasantly at sea, and who sprained his wrist playing leap-frog on deck the last day of my voyage.

Shrove Tuesday is a great time for practical jokes, especially whitening and blacking faces with flour or soot. I detected some of the younger servants egging each other on to assault me with the dredger; but I went on beating up my eggs quite unmoved, feeling pretty sure none dare venture really to play pranks on me. Sometimes of an evening a pretty little chambermaid brings a wire-strung guitar nearly as big as herself into my room, and plays and sings fandoes, irresistible dance tunes very like Scotch reels. With her comes the grey-haired old dame who looks after the children, and plays propriety for the maids; and every now and then, when her favourite airs sound, she leaps to her feet and capers till breathless.

Last Friday I had a lovely ride. A patient—not my own case, but one I have annexed—treated me. After a short tear along the New Road, the only level one in Madeira, the horses began to climb. Up and up the steep, straight hills they went at a rushing, scrambling trot; and coming down was much the same. They are as sure-footed as goats, and hardly need any driving. Our destination was a beautiful ravine about half-way up the mountain overlooking Funchal. Fine tall pines grow on its sides, and the air has something of that dry match-box-like smell we are told is so good for consumptives. It is very lonely—hardly a cottage till the head of the ravine is reached; there one looks right down on the whole town and country below. The country was at its brightest after the rain, corn greener than grass, with quantities of a crimson flower like a small gladiolus; acres of marigold, and peach and pear trees just smothered in pink and white. Nurse Dickenson.

### The Little One's Mite.

"I wis' I'd some money of my own," sighed a very little mortal, with wistful blue eyes shining out of a crowd of fair curls.

Seated on a small stool, in the flickering firelight, Elsie Charteris seemed to concentrate all the light there was upon her tiny person, the rest of the room being filled with dusky shadows. And so it was, in the wealthy home to which she belonged the child was the centre round which all sweetness and light gathered. She was the very pulse of old Granny's heart—Granny who had seen her dear ones slip out of life until all were gone home save herself and Elsie, the eldest and the youngest. Try as she did, Granny could not help setting her heart with passionate yearning on the small human fragment left of her large family, and Elsie would have been spoiled, if the child could spoil. Had Elsie cried for the stars, Granny would like to have raked them down from the skies for her to play with. And now, sitting knitting in the dusky room, the old lady hears the little one's audible wish.

"Money of your own, my child! What could you do with money? Can't I buy what you want—eh?"

"No, Granny, not quite esakly," and the fair little head went from side to side; "you see I've got a secret. Now, Kitty, don't wiggle." Kitty was not very comfortable, being wrapped tightly in Elsie's pinafore, but it is marvellous what pussies will bear at the hands of children.

"I don't think you can know what a secret means, my pet," and Granny smiled.

"Oh, but I do know, Gran; it's something wrapped up in a corner of my head, where nobody can see it."

"Very well, dear; keep it away out of poor old Granny's sight, if you can; but I can guess that the money you wish for is to buy a new doll."

"No, Granny; it isn't a doll. Guess again."

"Let me see, then. It is a set of doll's tea-things?"

"No, no!" cried Elsie, delighted with the guessing game.

"Dear, dear. Well, it must be doll's furniture. What else can a doll owner want? Perhaps it's a new bedstead for your best doll, Lady Annabella?"

"Oh-h-h!" Elsie almost screamed, and Kitty got such a squeeze that she mournfully squeaked. "You're dweadfully hot, Gran, just burning hot, but not esakly right either. Now, please, don't guess any more, or you'll see right into my head." Elsie jumped up, releasing the cat, to fling her little arms round Granny's neck, and just then the drawing room door opened to admit John with the tea-things; the lamps were lighted, the dim shadows fled away, and the fire sulked because it was extinguished. There was a tinkling of silver spoons against dainty china as Granny with little Elsie drank hot tea and ate toast in the close of the winter afternoon.

The long, cold days were past; there was a faint stirring as Nature turned in her sleep, and the east wind had much ado to keep back forward young buds. But in and about the stately house there were no little eager feet flying to greet the first snowdrop or the gay daffodils. There was only Granny to go out for a solitary airing in the big, lonely carriage once a day. Granny in the body, that is to say, for her heart was at home in the nursery, where, on her little white bed, lay Elsie all the days as well as all the nights.

Something had happened to the little one since the winter afternoon when she and Granny had played that guessing game; something too sad for Granny to bear. The little maid had sustained a grievous accident. Going out for a donkey ride on Sir Roger, something never to be explained came over that hitherto decorous animal, and he had bolted down the drive, stumbling midway, and, horror of horrors! began to kick out violently before any one could get near to extricate his helpless wee rider. When the injured child was carried home, and laid upon her little bed, serious hurt was found to have been done by Sir Roger. "Her back is much injured," said the mighty medicine man for whom they had telegraphed to London.

"Will she live?" gasped Granny in a strangled voice.

"Hum! She *may;* but I honestly tell you that will depend more upon your care than our help. We'll do all science has taught us; you must do the rest Watch her as a cat watches a mouse—that is, never let her out of your thoughts. Perhaps you may thus keep her life. Ah—*Good* morning!" and the great man bowed himself out.

What a battle followed between Granny and grim death! At last the victory was won, but the trophy was a mere human shadow of a child. Slowly, slowly health came creeping back, but not the power of movement, the spine being the injured part.

"She ought to lie in a mould," said the family doctor.

"I s'ant," said a thin small voice; "I ain't a jelly.

"Well done!" laughed the old man. "They haven't knocked the mother-wit out of you! Keep up a good heart," he added, turning to Granny. "She'll weather it yet, if I'm not mistaken," And the little one—with God's help—did weather it. That is, she lay still and white all the days waiting for healing to come.

"Elsie, my pet," said Granny, sitting by her bed side, "do you remember how I heard you wishing you'd lots of money of your own, once, last winter?"

"Of course I do, Gran, and you nearly guessed what I wanted to buy; you got very hot."

"Did I, darling? Well, you know, this is your birthday, and I want to give you what you will like best. See!" And Granny unclasped a morocco bag which was full of shining sovereigns. "You may help yourself to a handful of these."

"Thank you, Granny," and Elsie spoke soberly; "that is just the sort of pwesent I'd like best. But, first, will you please ask nurse for my money box?"

When the money-box was opened its owner counted out her money. Quite a little pile was on the gay coverlet. The funny thing about Elsie's treasure was that it consisted entirely of silver threepenny-bits, it having always been Granny's custom to lay aside for the little one those small coins. It took time and considerable attention on Granny's part to do the sum, as Elsie called it, of her possessions.

"Now," cried Elsie, "I've saved all that on purpose for the thing I'm going to buy, and the soveweigns shall be put with it."

"But am I not to know what it is?"

"Oh, yes, Gran. You guessed it was to be a doll's bedstead. Well, it is a kind of one. It's a cot in the Children's Hospital!" And Elsie bubbled over with feeble laughter. "Don't you wemember," she went on, "that clergyman who came to see you last summer to tell you stowies about the hospitals? I made up my mind then to save up and buy a cot, and, O, Gran! you will help me?"

And Elsie got her way, of course; and Granny christened the cot "The Little One's Mite." More than that, the day came, in time, when Elsie was able to go herself and see what *she* called the doll's bedstead—and the live doll in it.

### Everybody's Opinion.

[*Correspondence on all subjects is invited. No communications can be entertained if the name and address of the correspondent is not given, or unless one side of the paper only be written on.*]

### NURSING THE INSANE.

DAFFODIL writes: I notice with great satisfaction the article in your last issue, headed "Nursing the Insane," and the hope expressed in it that in a few years we may have in this country a regular system of training for nurses and attendants. I write feelingly on this subject, having myself suffered severely from the exercise of "tactless, untrained" energy. I have a horror of writing hysterically or in any way sensationally, but the bare facts ought to be known, and there are, perhaps, few persons who are quite in a position to state them. An individual breaks down from, we will say, over-work, over-excitement, or one of the causes incident to our modern civilisation. Nervous exhaustion and prolonged sleeplessness ensue, with, perhaps, morbid impulses, and ultimately a state of the nerves is induced in which physical sensations, notably those of light and sound, are very considerably intensified, amounting in my own case, and doubtless in all similar cases, to positive, almost unendurable, torture. Now for a patient in this condition to be treated in an asylum as at present conducted, has all the effect, though not the intention, of extreme cruelty. Medical men well know how sadly unfit for asylum life are many of the cases, which nevertheless they are compelled to send, simply because there exists no other provision, nothing between the ordinary home life, and these, in some respects and in some states of mind, really terrible places. In the state of nervous prostration above mentioned a loud tone is magnified to a threatening shout, and banging of heavy doors and ringing of large bells for meals, etc., are well calculated to increase the fearful irritation, while, if expression should in any way be given to the same, the patient stands an excellent chance of being transferred to the "refractory" ward, or of being, to say the least, severely reproved. Then, as to the arrangements during meals, reform is imperative. An elderly lady, herself a patient, said, in reply to a severe criticism of mine: "Yes, my dear, all you say is perfectly true, but we can do nothing, as we are entirely in the hands of the attendants; we can only look up and wait God's own good time." Then and there I mentally registered a vow that, given the opportunity, I would do what I could to hasten the "good time." Practically, there is small chance of appeal from ill treatment, as such patients are far too nervous and shaken to be able to complain coherently either to doctors or lunacy commissioners, so the remedy must be found in proper training and qualification. It would be unjust, as well as ungrateful, to omit to recognise the fact that there are attendants and attendants; to those who have intelligently and sympathetically cared, one can never cease to feel the deepest obligation. What is wanted seems to me to be some amount at least of cultured common sense, some ability to discriminate between cases and temperaments that differ, gentle firmness, and sympathy.

We sent the above to our Lunacy Commissioner, who writes: "Daffodil" does not say whether her experience was derived from a private or a public asylum. If a private one, it is a matter entirely for the proprietor and the patients' friends. If a public one, it must be remembered that the asylum is supported by the rates, and it is extremely probably that the ratepayers would grumble if too highly paid nurses and attendants were engaged. In the majority of cases, at least in those cases where the medical superintendent has power to engage and discharge attendants as may seem fit to him, the nursing staff is fairly efficient, and is for the most part in sympathy with the class of patients in the asylum. No doubt our county asylums now and then contain patients who feel the whole of their surroundings to be a grievance, and the slightest appearance of harshness is unintentionally magnified a hundredfold. For one such case there are ninety-nine who are in every respect much better off than they would be at home.

That the nursing in our Asylums is not wholly satisfactory is only saying that it is human, and the improvement would seem to lie in the better payment of the nurses generally, thus giving the medical superintendent a wider selection. But then an asylum of average size will contain sixty attendants and nurses, and to increase their annual wages by £5 would mean £300 a-year on to the expenditure. A cry for shorter hours of duty would soon arise, and perhaps four supernumeries would have to be added, meaning, with board and lodging, £180 a-year.

We believe these alterations would result in an improvement almost immediately, and the power to inaugurate this reform rests entirely with the Committee of Visitors. Let "Daffodil" procure the names of the visitors of the Asylum for her district. See what she can do to influence them, and send us the results. Merely writing to the papers is useless. Whether the improvement would be sufficient to justify the expenditure is a thing which experience would alone solve. Our own experience is not extensive enough to enable us to generalise from; but so far as it goes it would go to prove that governesses, "superior persons," and trained hospital nurses do not make good asylum nurses.

It has been proposed to place these "superior persons" in charge of the wards. We believe this has not yet been tried, but it would mean additional mess-rooms and sitting-rooms for the staff, and would seem hardly fair to the under nurses, as it would cut the hope of promotion from them, annihilating one reason for remaining in the service, and necessitating frequent changes in the staff.

As to their being no half-way house between the home and the asylum, we can only say that the difficulties of establishing these would in all cases be great, and in most cases simply impossible to get over. Hospitals for acute cases have been tried in other countries, and have not proved successful. They end, as a writer in the *British Medical Journal* lately said, by resembling the refractory wards in our own asylums. The smallest of our county asylums contains at least one ward which is nearly always perfectly quiet; and medical superintendents are only too anxious to get as many patients as possible out of the so-called Refractory. In none of our county asylums is a charge of ill-treatment ever passed by, and when substantiated, instant dismissal of the nurse is the rule.

The following points would seem to be certain:—

1st. That mental nurses can only be trained in asylums.

2nd. That nurses so trained, if good and sensible, will not voluntarily leave the asylum in which they have been trained.

3rd. That medical superintendents are practically unanimous that the nurses they get from other institutions leave all the "good" they learned, and bring only or chiefly the "bad."

4th. Every asylum must, therefore, train its own nurses, and to do this suitably and effectually, good raw material must be at hand.

5th. Much of the cry among women of the better class for suitable employment is a cry, and nothing more. It is the rarest thing for them to apply at our county asylums for employment, and it is rarer still for them to consent to work their way up.

---

### THE CARE OF THE INSANE.

T. A. WRITES: Whilst it is most desirable that, if possible, attendants on the insane should be of a better educated and more intelligent class of women than is at present the rule, would it be easy to find women of refinement willing to undertake asylum work? The demoralizing habits, profane and indelicate language which the asylum nurse has to see and hear would be too revolting for most women of refinement and education to endure, and, although we are well aware that these poor creatures are not answerable for their actions, still it would be beyond the power of many to submit to the gross insults you are so often subject to. If all attendants could receive some instruction in general nursing, I think it would be an advantage, for the ignorance of many on this subject is deplorable. I remember some years ago, at an asylum in the West of England, a newly-appointed head attendant, on making inquiries, found only one nurse out of the twenty-eight on the staff who had any knowledge of the use of the clinical thermometer. Employment for the patients is to some extent a means of treatment, and in a public asylum the patients are usually from a class accustomed to domestic work, and consequently more likely to take kindly to it than to other employment; therefore, it seems to me that the attendant should be able to superintend practically, if necessary, the work performed by the patients under her care.

### Appointments.

[It is requested that successful candidates will send a copy of their applications and testimonials, with date of election, to THE EDITOR, The Lodge, Porchester Square, W.]

BURY HOSPITAL FOR INCURABLES.—Miss Frances M. Galloway, at present matron of the Workington Infirmary, has been appointed matron of the Bury Branch of the Northern Counties Hospital for Incurables. Miss Galloway trained at Edinburgh Infirmary, and was afterwards a staff nurse at the Longmore Hospital for Incurables, Edinburgh. Miss Galloway possesses excellent testimonials, and is eminently fitted for the post she has received.

### Vacancies.

The following vacancies are announced:—

**Matrons.**

Victoria Cottage Hospital, Barnet; £30.

Wrexham Fever Hospital; £30.

Princess Alice Memorial Hospital; £50.

**Head Nurse.**

Brighton Borough Fever Hospital; £28.

**Nurses.**

City of London Hospital; £20.

Hampstead Home Hospital.

Deaconesses' House, Chester; £24.

Southport Nurses' Home.

West Kensington Nursing Home.

Nursing Home, Buckingham.

Kingston Home, Hull.

Tunbridge Wells Hospital; £25.

Public Hospital, Sheffield; £22.

Nurses' Home, Newcastle-on-Tyne; £25.

Nurses' Institute, Jersey; £25.

Nurses' Home, Leicester.

Bridgnorth Union; £25.

Kingston Nurses' Home, Hull; several.

St. Pancras Infirmary, Highgate; several; £20.

Royal Albert Hospital, Devonport: several; £25.

Eastbourne Nursing Institution.

St. George's Home, Sheffield.

Workhouse Infirmary Nursing Association.

Crescent-road, Beckenham.

Notts Nursing Institution; £25.

Ryde Nursing Institution.

Levick Paris Institution.

Swansea Nursing Institution; £25.

Nursing Institution, 96, 97, 98*a*, Wimpole Street, London, W., Mr. Wilson has several vacancies for thoroughly-trained Nurses.

Brighton Borough Fever Hospital; two; £24.

Holborn Union; two; £20.

Kent Nursing Institution; several.

Belvedere Hospital, Glasgow.

**Pupil Nurses.**

Taunton and Somerset Hospital; several.

**District Nurses.**

Corbridge-on-Tyne; £75.

Shalford; £26.

Watford.

Brighton; £35.

Madeley (Staffs).

**Ward Assistants.**

St. Pancras Infirmary, Highgate; several; £14.

**Probationers.**

Belvedere Hospital, Glasgow; several.

Kent Nursing Institution.

Royal Hospital for Diseases of the Chest; £12.

### Notes and Queries.

**Queries.**

*Wanted Needlework.*—Will anyone give needlework to a gentlewoman who, through ill-health, cannot take a situation, and has an aged aunt without any means to support.—*K. B.*, The Red House, Portslade-by-Sea, Brighton.

(138.) *Nursing in France.*—Can anyone tell me where I should find a good opening for independent private nursing in the South of France, and how a start should be made?—*F. M.*

(139.) *Pronouncing Dictionary.*—Can you tell me of a pronouncing medical dictionary, and what it would cost?—*Private Nurse.*

(141.) *Papier Gondronne.*—Where can it be obtained?—*Amateur Nurse.*

**Answers.**

(137.) *Nurse Groom.*—Apply to our publisher at 130, Salisbury Court, he will send you the numbers you require. Get *Misericordia*, price 2d., from Knott, 20, Brooke Street, Holborn, for information concerning the guild of St. Barnabas.

(111.) *Register for Midwives.*—There is such a register at 30A, Bold Street, Liverpool.—*Nurse Ellen.*

(134.) *Domestic Medicine.*—A new edition of this little handbook has just been published, and can be got through any bookseller for 1s.

(134.) *Domestic Medicine.*—Dr. W. J. Russell has published a book so called, price 2s.

(142.) *Dilemma.*—Ambulance classes are held at Brighton. Apply to the local secretary of the St. John's Ambulance Association. Probationers must be 21 years old for local hospitals; 25 for London hospitals.

(140.) *College of Surgeons' Museum.*—New Wandsworth Nurses are informed that they can see the Hunterian Museum on the first four days of the week, between eleven and five, by presenting your card. The other parts of the museum can only be seen in company with a member of the College, or as a special favour.

*Troubles at Stroud.*—The Secretary of the Stroud Hospital is anxious his institution should not be confounded with Stroud Road Hospital, mentioned last week.

*L. A. W.*—None in London. Dr. Goodhart, Mr. Jacobson.

---

*Address Wanted.*—Will A. E. S. please send her address? It has been mislaid, and there is a letter waiting for her.—*Editor.*

